# Innovations and Outcomes in Astigmatism Correction During Cataract Surgery: A Comprehensive Review

**DOI:** 10.7759/cureus.67828

**Published:** 2024-08-26

**Authors:** Vijaya Mallareddy, Sachin Daigavane

**Affiliations:** 1 Ophthalmology, Jawaharlal Nehru Medical College, Datta Meghe Institute of Higher Education and Research, Wardha, IND

**Keywords:** surgical innovations, visual acuity, femtosecond laser-assisted cataract surgery (flacs), toric intraocular lenses (iols), cataract surgery, astigmatism correction

## Abstract

Astigmatism, a prevalent refractive error caused by an irregular curvature of the cornea or lens, can significantly affect visual acuity and the quality of life. Correcting astigmatism during cataract surgery is essential for achieving optimal postoperative visual outcomes. This comprehensive review examines recent innovations in astigmatism correction methods and their impact on cataract surgery. It provides an in-depth analysis of advancements such as toric intraocular lenses (IOLs), femtosecond laser-assisted cataract surgery (FLACS), and new IOL technologies designed to address astigmatism with greater precision. The review also evaluates clinical outcomes, including visual acuity improvements, patient satisfaction, and safety considerations associated with these innovations. Additionally, it explores the cost-effectiveness of various techniques and highlights emerging trends and future directions in the field. By synthesizing current evidence, this review aims to offer valuable insights for clinicians and inform best practices in astigmatism management during cataract surgery.

## Introduction and background

Astigmatism is a common refractive error characterized by an uneven curvature of the cornea or lens, resulting in distorted or blurred vision. Unlike spherical refractive errors, where light is focused on a single point on the retina, astigmatism causes light to be focused on multiple points, either in front of or behind the retina [1]. This leads to difficulties seeing clearly at various distances and can affect overall visual quality. Astigmatism often coexists with other refractive errors such as myopia (nearsightedness) or hyperopia (farsightedness). It is a prevalent condition affecting a significant portion of the population, with estimates suggesting that approximately 30% of adults have some degree of astigmatism [2].

Cataract surgery, a common procedure for removing and replacing a cloudy lens with a clear artificial lens, often requires the careful management of pre-existing astigmatism to optimize visual outcomes. Uncorrected astigmatism can significantly impact postoperative visual acuity and patient satisfaction [3]. The effective correction of astigmatism during cataract surgery is crucial for achieving optimal visual outcomes and enhancing patients' overall quality of life. Traditionally, astigmatism correction has been addressed through various surgical techniques and intraocular lenses (IOLs), but recent advancements have provided new options for improving precision and effectiveness [4].

This review aims to explore and summarize the latest innovations in astigmatism correction during cataract surgery. Recent developments in surgical techniques, IOL technology, and procedural integration have expanded the options for managing astigmatism. Innovations such as toric intraocular lenses, femtosecond laser-assisted cataract surgery (FLACS), and new IOL technologies have enhanced accuracy and improved visual outcomes. By examining these advancements, the review aims to provide a comprehensive overview of how they have transformed astigmatism management in the context of cataract surgery.

## Review

Astigmatism in cataract surgery

Astigmatism is a prevalent refractive error that can significantly influence the outcomes of cataract surgery. It is characterized by an irregular curvature of the cornea or lens, resulting in blurred or distorted vision. A thorough understanding of the different types of astigmatism is essential for accurate diagnosis and effective treatment [3]. Astigmatism is primarily classified into two types: corneal and lenticular. Corneal astigmatism can be further subdivided into regular and irregular types. Regular corneal astigmatism occurs when the cornea maintains a consistent curvature but is shaped more like a football than a sphere, causing light to focus at multiple points [5]. This type is often described by principal meridians perpendicular to each other. In contrast, irregular corneal astigmatism arises when the cornea's curvature varies in different directions, often due to conditions such as keratoconus, injury scarring, or postsurgical changes. In these cases, the principal meridians are not at right angles, leading to more complex visual distortions. On the other hand, lenticular astigmatism originates from irregularities in the lens, often associated with cataracts. As the lens becomes distorted, it contributes to blurred vision similar to that caused by corneal astigmatism [6].

Astigmatism is typically diagnosed through various methods, including keratometry and topography. Keratometry measures the cornea's curvature to assess the degree of astigmatism. At the same time, topography provides a detailed map of the corneal surface, allowing for the precise identification of irregularities and the extent of astigmatism [7]. The presence of astigmatism can have a substantial impact on cataract surgery outcomes, affecting both visual acuity and patient satisfaction. Residual astigmatism after cataract surgery can result in blurred vision, particularly at specific distances. Research indicates that correcting astigmatism during cataract surgery can improve visual acuity. For example, toric intraocular lenses (IOLs), specifically designed to address astigmatism, can significantly enhance the likelihood of achieving optimal refractive outcomes. Patient satisfaction is closely tied to visual outcomes, and when astigmatism is not adequately corrected during cataract surgery, patients may experience dissatisfaction due to persistent visual disturbances. Conversely, the successful correction of astigmatism can lead to higher satisfaction rates, as patients often report improved clarity and reduced dependence on corrective lenses following surgery [8].

Innovative techniques in astigmatism correction

Preoperative Assessment Innovations

The cornerstone of effective astigmatism correction is a comprehensive preoperative assessment. Advanced imaging techniques, such as optical coherence tomography (OCT) and corneal topography, have transformed how surgeons evaluate the cornea. OCT provides high-resolution cross-sectional eye images, enabling a detailed analysis of the corneal structure and any irregularities. Conversely, corneal topography maps the cornea's curvature, offering critical insights into the nature and degree of astigmatism. These imaging modalities work together to allow surgeons to customize their surgical plans to meet each patient's needs, thereby ensuring more precise outcomes [9]. Beyond imaging, advanced biometric measurements have become indispensable in preoperative evaluations. State-of-the-art biometric devices, such as the IOLMaster 700 and Lenstar, deliver precise measurements of key ocular dimensions, including keratometry and axial length. These measurements are vital for accurate intraocular lens (IOL) power calculations, which are directly linked to the success of astigmatism correction. By leveraging these advanced tools, surgeons can significantly enhance the accuracy of their surgical interventions [10].

Surgical Techniques

Innovative surgical techniques have significantly advanced the correction of astigmatism during cataract surgery. Among the most notable developments is the use of toric intraocular lenses (IOLs). These specialized lenses are designed to correct astigmatism and come in single-piece and multi-piece configurations. Single-piece toric IOLs generally offer enhanced stability, while multi-piece options provide greater flexibility in positioning [11]. Recent advancements in toric IOL technology have expanded their capabilities, allowing for the correction of higher levels of astigmatism: up to 6.00 diopters (D) at the IOL plane. Additionally, some of the latest toric IOLs are designed to permit postoperative adjustments, further improving the precision of astigmatism correction [12]. Another significant innovation is the introduction of laser-assisted cataract surgery, particularly with femtosecond laser technology. This technology enables surgeons to create highly precise corneal incisions tailored to the individual patient's astigmatism. By enhancing the accuracy and reproducibility of surgical interventions, femtosecond lasers reduce the potential for human error and improve overall surgical outcomes [13].

Traditional surgical techniques, such as keratotomy and relaxing incisions, remain relevant in astigmatism correction. Methods such as limbal relaxing incisions (LRI) and peripheral corneal relaxing incisions (PCRIs) are effective, especially when combined with toric IOLs. Modern approaches use advanced imaging to guide these incisions more accurately, improving the predictability of results. Combining relaxing incisions with toric IOLs can significantly enhance the overall correction of astigmatism, particularly in patients with higher degrees of pre-existing astigmatism [14]. Corneal surgery techniques, such as astigmatic keratotomy (AK), also play a valuable role. AK involves making precise incisions in the cornea to flatten the steep meridian, thereby reducing astigmatism. This technique can be performed with cataract surgery, providing a comprehensive approach to astigmatism correction [15]. A comparison of innovative techniques for astigmatism correction during cataract surgery is shown in Table 1.

**Table 1:** A comparison of innovative techniques for astigmatism correction during cataract surgery PRK, photorefractive keratectomy; LASIK, laser-assisted in situ keratomileusis

Technique	Description	Advantages	Challenges
Toric intraocular lenses (IOLs)	Specialized lenses are designed to correct astigmatism by being implanted during cataract surgery.	High accuracy in correcting astigmatism and long-term stability.	It requires precise alignment and has a higher cost compared to standard IOLs.
Femtosecond laser-assisted cataract surgery (FLACS)	Utilizes femtosecond laser technology for precise corneal incisions tailored to correct astigmatism.	Enhanced precision and reduced human error.	Costly and requires specialized equipment and training.
Limbal relaxing incisions (LRI)	Incisions made in the cornea to reduce astigmatism are often combined with cataract surgery.	Simple and it can be effective for mild astigmatism.	Less predictable outcomes, particularly for higher astigmatism.
Peripheral corneal relaxing incisions (PCRIs)	Similar to LRI, it is performed on the peripheral cornea to correct astigmatism.	Effective for low levels of astigmatism and minimally invasive.	Predictability decreases with higher astigmatism.
Astigmatic keratotomy (AK)	Corneal surgery involves precise incisions to flatten the steep meridian of the cornea.	It can be combined with cataract surgery for comprehensive correction.	The potential for regression requires a precise technique.
Custom ablation techniques	Laser-based procedures (e.g., PRK and LASIK) tailored to the individual's corneal topography.	Highly customizable and it can correct a range of astigmatism levels.	It is expensive and requires advanced imaging and planning.
Intraoperative aberrometry	Real-time measurement of the eye's refractive status during surgery to optimize IOL placement.	Improves the accuracy of surgical outcomes and real-time adjustments.	It requires specialized equipment and adds complexity to the procedure.

Outcomes of astigmatism correction innovations

Visual Outcomes

One of the most significant advancements in astigmatism correction has been the improvement of refractive stability and accuracy, particularly through toric intraocular lenses (IOLs). These specialized lenses are designed to correct astigmatism, offering a predictable reduction in refractive error [16]. Clinical studies have shown that patients who receive toric IOLs often achieve postoperative residual astigmatism of 0.5 diopters (D) or less, which is considered optimal for visual acuity. For example, research indicates that patients undergoing customized ablation photorefractive keratectomy (PRK) experience a significant reduction in astigmatism, with an average decrease of -1.67 D, from a preoperative average of -2.51 D to -0.87 D postoperatively [17]. Similarly, laser-assisted in situ keratomileusis (LASIK) procedures for correcting myopic astigmatism have resulted in high rates of uncorrected distance visual acuity (UDVA), with approximately 90% of patients achieving 20/20 vision or better within a year after surgery [18]. The comparison of preoperative with postoperative visual acuity further highlights the effectiveness of these innovations. Patients undergoing cataract surgery with toric IOLs often report significant improvements in visual clarity and a reduced dependence on corrective lenses. Achieving a UDVA of 20/25 or better is a common outcome, demonstrating the success of modern correction techniques in enhancing overall visual performance [19].

Patient Satisfaction

In addition to the technical advancements in visual outcomes, patient satisfaction has significantly improved following astigmatism correction during cataract surgery. Many patients report marked improvements in their quality of life, highlighting the newfound ability to engage in daily activities without relying on glasses or contact lenses. The strong correlation between reduced astigmatism and enhanced quality of life is well-documented, with patients expressing satisfaction in various aspects of their lives, such as driving, reading, and participating in social activities [20]. Patient-reported outcomes, often gathered through satisfaction surveys, indicate high levels of contentment with the results of astigmatism correction. A substantial majority of patients feel that their expectations have been met or even exceeded, especially when toric IOLs are used. This positive feedback is largely due to reduced visual disturbances and the liberation from the need for corrective eyewear, significantly enhancing overall daily functioning [21].

Complications and Risks

Despite the advancements in astigmatism correction techniques, potential complications can still occur during and after surgery. Intraoperative risks include the incorrect positioning of toric IOLs and the potential for corneal damage during the creation of incisions. Postoperatively, patients may experience residual astigmatism, which can adversely affect visual outcomes if not appropriately addressed [4]. To mitigate these risks, surgeons employ various risk management strategies. Careful preoperative planning, often supported by advanced imaging technologies such as corneal topography and optical coherence tomography (OCT), is crucial. These tools provide precise measurements and aid in accurately selecting IOLs, thereby minimizing the chances of complications. Additionally, the continuous monitoring of surgical outcomes allows for technique and approach adjustments based on prior cases' empirical data, ensuring that patients receive the highest standard of care [22].

Comparative analysis of techniques

Effectiveness

When assessing the effectiveness of various astigmatism correction methods, toric intraocular lenses (IOLs) emerge as the most successful option. Extensive studies have shown that patients receiving toric IOLs achieve superior uncorrected distance visual acuity (UDVA) and significantly lower levels of residual astigmatism compared to those undergoing femtosecond laser-assisted astigmatic keratotomy (FSAK) [19,23-27]. For instance, a meta-analysis reported that the mean residual refractive astigmatism in patients with toric IOLs was approximately -0.63 diopters (D). In contrast, those who underwent FSAK had a mean residual astigmatism of -0.90 D three months post surgery. This evidence highlights the reliability and predictability of toric IOLs in effectively managing astigmatism [25]. In contrast, while FSAK can reduce astigmatism, its outcomes are generally less predictable, especially for higher degrees of astigmatism. The long-term stability of the results from FSAK also falls short compared to toric IOLs. Similarly, peripheral corneal relaxing incisions (PCRIs) can effectively correct low levels of astigmatism, but their predictability decreases as the severity of astigmatism increases. Overall, toric IOLs are widely regarded as the gold standard for astigmatism correction during cataract surgery due to their superior outcomes [28]. Long-term outcomes further underscore the advantages of toric IOLs. Research shows that patients with toric IOLs maintain better visual acuity and lower levels of astigmatism years after surgery than those who undergo other correction methods. This durability makes toric IOLs the preferred method for surgeons and patients [29].

Cost-Effectiveness

Cost-effectiveness is a crucial factor when evaluating astigmatism correction techniques. A recent analysis found that while the implantation of toric intraocular lenses (IOLs) incurs a higher societal cost of approximately €3,203 compared to €2,796 for monofocal IOLs, the quality-adjusted life years (QALYs) gained are relatively similar between the two groups. This finding suggests that toric IOLs may be less cost-effective from a societal perspective, raising concerns about their economic viability, especially in healthcare systems with limited resources [30]. The economic impact of these higher costs may lead to the necessity of patient copayments for toric IOLs, particularly given the marginal benefits in QALYs compared to less expensive alternatives. Consequently, healthcare systems must carefully consider the trade-offs between the benefits of improved visual outcomes and the financial implications of toric IOLs [31].

Technological Advancements

**Figure 1:**
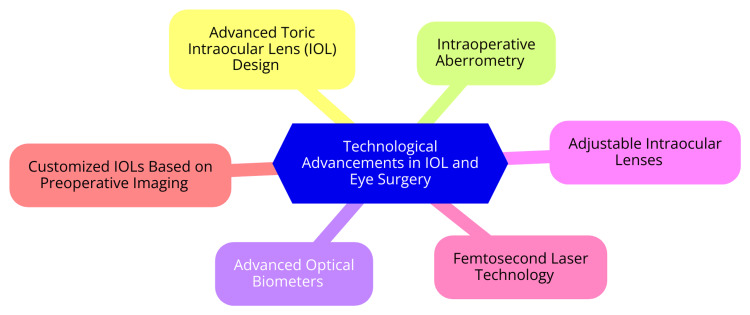
Technological advancements in IOL and eye surgery Image credit: Dr. Vijaya Mallareddy IOL: intraocular lens

Technological advancements have significantly enhanced the effectiveness of astigmatism correction techniques. Recent innovations in toric intraocular lens (IOL) design focus on expanding the range of astigmatism that can be corrected and improving the postimplantation stability of these lenses. These new designs aim to reduce the impact of preoperative measurement errors, which can lead to suboptimal outcomes. Additionally, advancements in intraoperative aberrometry now allow for real-time measurements during surgery, greatly increasing the accuracy of lens power selection and placement [4]. Emerging technologies, such as advanced optical biometers, are also revolutionizing preoperative assessment. These devices provide precise measurements that enable surgeons to customize their approach to meet each patient's needs. Future innovations may include the development of adjustable IOLs and more sophisticated surgical techniques, which could further enhance patient outcomes and broaden the range of candidates eligible for effective astigmatism correction [32]. Figure 1 illustrates these technological advancements.

Clinical guidelines and recommendations

Current guidelines from major ophthalmic societies, including the American Academy of Ophthalmology (AAO), European Society of Cataract and Refractive Surgeons (ESCRS), International Council of Ophthalmology (ICO), and All India Ophthalmological Society (AIOS), provide a robust framework for best practices in cataract surgery with astigmatism correction. These guidelines are designed to enhance patient outcomes and safety through evidence-based approaches [33]. The AAO's Preferred Practice Patterns (PPP) offer a comprehensive set of guidelines that cover various aspects of eye care, including cataract surgery and astigmatism correction. These guidelines emphasize the importance of accurate preoperative assessments, appropriate patient selection, and the use of advanced technologies to achieve optimal surgical outcomes. The ESCRS guidelines focus on preventing complications such as infectious endophthalmitis during cataract surgery, with regular updates to incorporate new research and techniques [34].

The ICO provides international clinical guidelines to improve the quality of eye care worldwide. These guidelines address conditions such as diabetic eye care and uncorrected refractive errors, stressing the need for comprehensive assessments and appropriate interventions. The AIOS has developed guidelines tailored to Indian healthcare, focusing on preoperative, intraoperative, and postoperative measures to minimize risks during cataract surgery. These include detailed checklists to ensure adherence to best practices, particularly in resource-limited settings [35]. A comprehensive preoperative assessment is crucial to best practices in astigmatism correction during cataract surgery. This assessment includes a thorough ophthalmic evaluation to identify potential risk factors for complications, such as systemic health issues and ocular conditions that could affect surgical outcomes. The accurate measurement of refractive errors, including astigmatism, is essential and often involves advanced diagnostic tools such as corneal topography and optical coherence tomography (OCT). These tools ensure precise measurements that inform the choice of correction techniques. Additionally, a detailed patient history, including information on previous ocular surgeries and current medications, should be collected to tailor the surgical approach effectively [36].

The choice of astigmatism correction technique should be based on individual patient factors. For patients with significant astigmatism, toric intraocular lenses (IOLs) are often recommended due to their superior visual outcomes compared to standard monofocal IOLs. Techniques such as arcuate keratotomy or femtosecond laser-assisted procedures may be considered in lower astigmatism or specific corneal profiles. The decision should also consider the patient's lifestyle, visual demands, and preferences regarding spectacle independence [37]. Adhering to these guidelines and best practices enables ophthalmologists to optimize surgical outcomes and enhance patient satisfaction in cataract surgery with astigmatism correction. By following these evidence-based frameworks, clinicians can ensure that each patient receives personalized treatment tailored to their unique needs and preferences, delivering high-quality eye care [38]. Clinical guidelines and recommendations for astigmatism correction during cataract surgery are summarized in Table 2.

**Table 2:** Clinical guidelines and recommendations for astigmatism correction during cataract surgery OCT, optical coherence tomography; IOLs, intraocular lenses

Guideline/organization	Focus area	Key recommendations
American Academy of Ophthalmology (AAO)	Preferred Practice Patterns (PPP) for cataract surgery	Comprehensive preoperative assessment, including the measurement of refractive errors and astigmatism. The use of toric IOLs for significant astigmatism. Postoperative follow-up to assess outcomes.
European Society of Cataract and Refractive Surgeons (ESCRS)	Cataract surgery guidelines	Emphasis on preventing complications such as infectious endophthalmitis. Advanced imaging techniques are used for precise astigmatism correction.
International Council of Ophthalmology (ICO)	International clinical guidelines for eye care	The comprehensive assessment and treatment of refractive errors, including astigmatism. Emphasis on global standards and tailored interventions based on patient needs.
All India Ophthalmological Society (AIOS)	Best practices in cataract surgery in the Indian context	Detailed preoperative evaluation, including refractive error measurement. The use of toric IOLs for astigmatism correction. Adherence to checklists for minimizing risks during surgery.
General best practices	Tailored astigmatism correction based on patient factors	The use of advanced diagnostic tools (OCT and corneal topography) for accurate measurement. The choice of correction technique is based on the patient's lifestyle and visual demands.

Future directions

The future of astigmatism correction during cataract surgery is on the brink of significant advancements fueled by innovative research and emerging technologies. Among the most promising developments are light adjustable lenses (LALs), which can be fine-tuned postoperatively using ultraviolet light to optimize vision. This adaptability is particularly advantageous for patients with varying degrees of astigmatism. Additionally, the increasing use of femtosecond laser technology allows for the creation of highly precise corneal incisions, improving the effectiveness of techniques such as arcuate keratotomy. Intraoperative aberrometry is another exciting advancement, enabling the real-time measurement of the eye's refractive status during surgery and allowing surgeons to make immediate adjustments for optimal outcomes [23]. Advances in personalized medicine are also paving the way for more tailored approaches to astigmatism correction. Research into the genetic basis of refractive errors may lead to individualized treatment plans considering an individual's unique ocular characteristics and family history. Furthermore, developing customized intraocular lenses based on detailed preoperative imaging and patient-specific refractive profiles holds great promise for achieving more precise astigmatism correction. Enhanced preoperative evaluation techniques, such as swept-source optical coherence tomography (OCT), provide more detailed assessments of corneal topography and anterior segment anatomy, informing personalized surgical strategies [39]. Despite these promising advancements, several challenges persist in astigmatism correction. One major issue is the occurrence of residual astigmatism, with some patients still experiencing uncorrected astigmatism after surgery, which may require enhancement procedures or additional treatments. Additionally, innovative treatments such as personalized IOLs and advanced imaging techniques can be cost-prohibitive and may not be widely accessible, highlighting the need for broader implementation and insurance coverage. Ensuring surgeons are adequately trained to use new techniques and technologies effectively is crucial for maximizing patient outcomes [40]. Future directions in astigmatism correction during cataract surgery are detailed in Table 3.

**Table 3:** Future directions in astigmatism correction during cataract surgery OCT: optical coherence tomography

Future direction	Description	Potential benefits	Challenges
Light adjustable lenses (LALs)	Lenses that can be fine-tuned postoperatively using ultraviolet light to optimize vision.	Allows for precise adjustments after surgery, improving visual outcomes, particularly for varying degrees of astigmatism.	Cost, availability, and ensuring precise adjustment protocols.
Femtosecond laser technology	Enhanced use of femtosecond lasers to create precise corneal incisions for procedures such as arcuate keratotomy.	Increases the precision of surgical incisions, potentially improving the effectiveness of astigmatism correction techniques.	High costs, need for advanced training, and accessibility in different regions.
Intraoperative aberrometry	Real-time measurement of the eye's refractive status during surgery, enabling immediate adjustments.	Enhances the accuracy of lens placement and power, leading to better visual outcomes.	Technical complexity and additional time required during surgery.
Personalized intraocular lenses (IOLs)	The development of customized IOLs based on detailed preoperative imaging and patient-specific refractive profiles.	More precise astigmatism correction tailored to individual patient needs leads to improved patient satisfaction.	Expensive development, manufacturing, and limited access to personalized lenses.
Genetic research and personalized medicine	Exploring the genetic basis of refractive errors to develop personalized treatment plans considering individual ocular characteristics and family history.	Tailored approaches to astigmatism correction, potentially leading to more effective and long-lasting outcomes.	Complex genetic profiling, ethical considerations, and the high cost of personalized treatment plans.
Enhanced preoperative evaluation techniques	Use advanced imaging techniques such as swept-source OCT for more detailed assessments of corneal topography and anterior segment anatomy.	Provides more accurate data to inform surgical strategies, potentially improving outcomes in complex cases.	It requires specialized equipment and training and may increase preoperative evaluation time and costs.

## Conclusions

In conclusion, advancements in astigmatism correction during cataract surgery have markedly improved patient outcomes and overall visual quality. Innovations such as toric intraocular lenses, femtosecond laser-assisted techniques, and advanced IOL technologies have significantly enhanced the precision and effectiveness of astigmatism management. These developments have led to better visual acuity, greater patient satisfaction, and reduced postoperative complications. As technology evolves, ongoing research and clinical trials will be essential in refining these methods and addressing any remaining challenges. By integrating these innovations into clinical practice, ophthalmologists can offer patients more effective and personalized treatment options, ultimately improving the quality of life for those undergoing cataract surgery.
